# Case Report: Synaptophysin-positive SMARCA4-deficient undifferentiated thoracic tumour: a diagnostic pitfall with therapeutic implications

**DOI:** 10.3389/fonc.2026.1871202

**Published:** 2026-06-05

**Authors:** Chang-Sen Bai, Xiong-Wen He, Shu Lin, Bai-Cheng Xu, Qi-Xing Yan, Ming-Fa Wang, Yue-Can Zeng, Jing-Ru Luo, Wen-Jun Tang

**Affiliations:** 1 Department of Radiation Oncology, Cancer Treatment Centre, The Second Affiliated Hospital of Hainan Medical University, Haikou, China; 2 Department of Oncology, Cancer Treatment Centre, The Second Affiliated Hospital of Hainan Medical University, Haikou, China; 3 Department of Pharmacy, The Second Affiliated Hospital of Hainan Medical University, Haikou, China; 4 Department of Pathology, The Second Affiliated Hospital of Hainan Medical University, Haikou, China

**Keywords:** case report, diagnostic pitfall, gene regulation, SMARCA4-UT, Syn

## Abstract

SMARCA4-deficient undifferentiated thoracic tumour (SMARCA4-UT) is a rare and highly aggressive malignancy with a poor prognosis and no established standard therapy. Isolated synaptophysin (Syn) expression may further complicate its differential diagnosis, particularly from neuroendocrine tumours. We report a 43-year-old male smoker with metastatic SMARCA4-UT, characterised by complete loss of SMARCA4/BRG1, a high Ki-67 index, and isolated Syn positivity, whereas other neuroendocrine markers were negative. The patient achieved a durable partial response lasting more than 12 months after platinum-based chemoimmunotherapy. This case highlights the potential clinicopathologic relevance of isolated Syn positivity in SMARCA4-UT. In this setting, isolated Syn positivity is more likely to reflect limited lineage de-repression rather than true neuroendocrine differentiation, and it may be associated with meaningful clinical benefit from chemoimmunotherapy. Isolated Syn positivity in SMARCA4-UT may represent a clinicopathologically relevant phenotype with potential therapeutic implications. Further studies are needed to clarify its diagnostic value and its possible association with treatment response.

## Introduction

SMARCA4-deficient undifferentiated thoracic tumour (SMARCA4-UT) is a rare and highly aggressive thoracic malignancy that was formally recognized as a distinct entity in the fifth edition of the World Health Organization (WHO) Classification of Thoracic Tumours in 2021. It occurs predominantly in male smokers and is characterized by rapid clinical progression, with most patients presenting with locally advanced or metastatic disease at diagnosis (1). Pathologically, the tumour typically shows undifferentiated or rhabdoid morphology together with loss of SMARCA4 expression (2). The prognosis is poor, with previous studies reporting a median overall survival of approximately 4.8 to 7.3 months (1).

Despite increasing recognition in recent years, SMARCA4-UT remains diagnostically and therapeutically challenging. Histologically, it is highly undifferentiated and may be difficult to distinguish from other poorly differentiated or undifferentiated thoracic malignancies, especially in small biopsy specimens (3). Notably, synaptophysin (Syn) expression has been reported in 60% of cases of SMARCA4-UT, which further increases the complexity of differential diagnosis with neuroendocrine tumours, especially large cell neuroendocrine carcinoma (LCNEC) (4). Currently, there is no standard treatment for SMARCA4-UT. Retrospective studies have suggested that SMARCA4-UT patients have limited benefit from traditional chemotherapy, and combination therapy based on immune checkpoint inhibitors (ICI) may achieve more durable remission (5).

Here, we report a case of advanced SMARCA4-UT with diffuse Syn expression that presented a major diagnostic challenge, mimicking a neuroendocrine neoplasm. The patient achieved a durable response after treatment with nab-paclitaxel, carboplatin, and a programmed cell death protein 1 (PD-1) inhibitor. This case highlights the potential clinicopathologic relevance of Syn positivity in SMARCA4-UT and raises the question of whether this phenotype may be associated with distinct therapeutic behaviour.

## Case presentation

A 43-year-old man was admitted with a 2-month history of low back pain accompanied by numbness in both lower extremities. He had a history of long-term heavy smoking and alcohol use, as well as chronic hepatitis C virus infection with mild hepatic dysfunction. On admission, physical examination revealed neurologic signs involving both lower extremities. His baseline ECOG PS was 2.

A baseline contrast-enhanced CT scan revealed a primary mass measuring approximately 40 × 33 mm in the upper lobe of the right lung. In addition to the primary lesion in the lung, multiple enlarged lymph nodes were identified in the bilateral neck, supraclavicular, subclavian, mediastinum, right hilum, and bilateral iliac. Immunohistochemically, the tumour showed focal CK(pan) and TTF-1 expression, diffuse synaptophysin positivity, a Ki-67 index of approximately 60%, retained Rb expression, focal INI-1 retention, and complete loss of BRG1/SMARCA4 expression. CK5/6, p40, CK7, Napsin A, CD56, chromogranin A, INSM1, PSA, GATA3, vimentin, and p53 were negative. Molecular testing did not identify common actionable driver alterations, and the programmed death ligand 1 (PD-L1) tumour proportion score (TPS) was <1%. Based on the undifferentiated morphology, complete BRG1/SMARCA4 loss, and the clinical-radiological context, the diagnosis of SMARCA4-UT was established, with clinical stage T4N3M1c and stage IVB.

**Figure 1:**
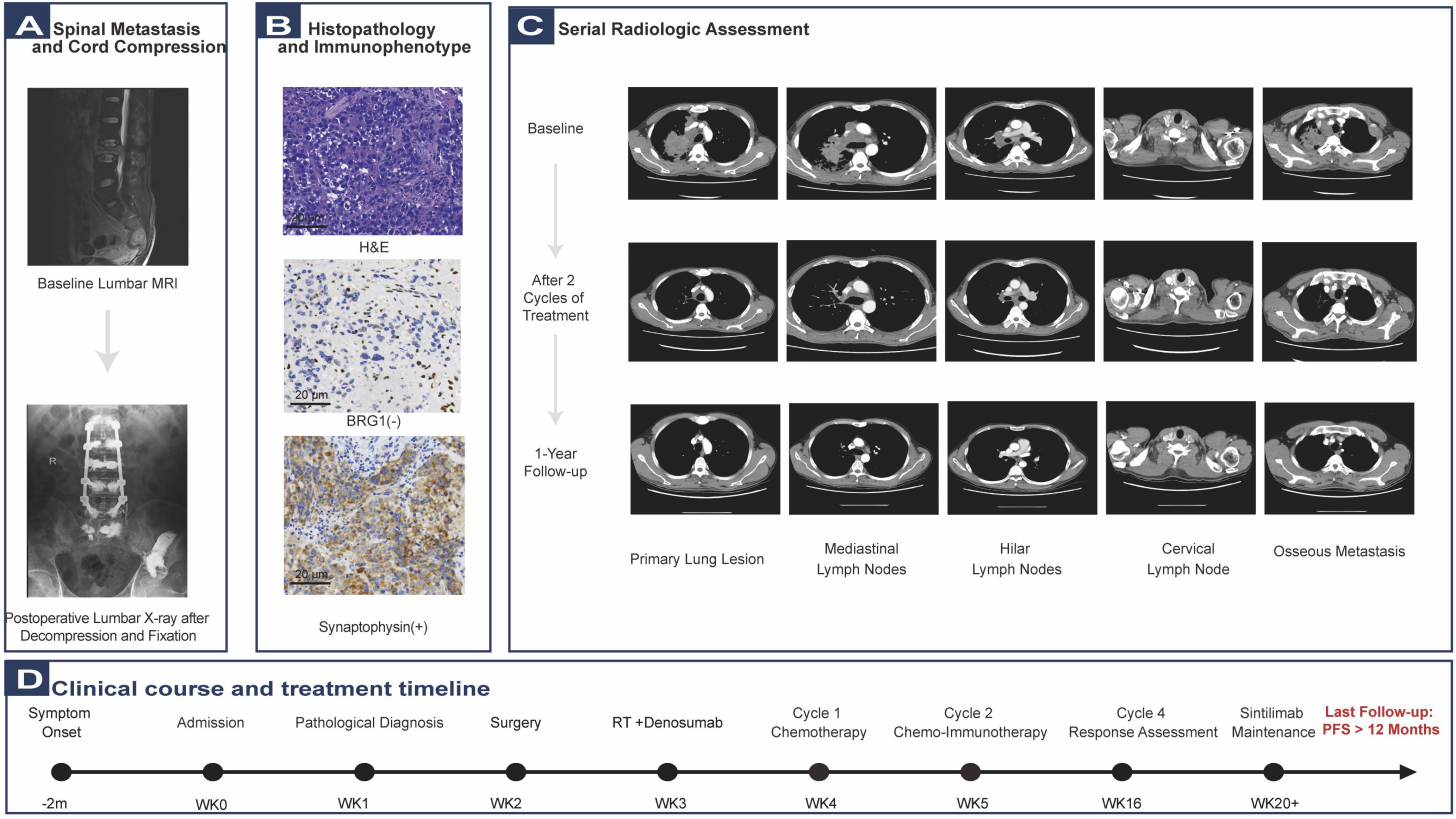
Clinical course, pathological findings, and radiological response in a patient with SMARCA4-deficient undifferentiated thoracic tumour. **(A)**, Baseline lumbar magnetic resonance imaging showed spinal metastasis with cord compression. Postoperative lumbar X-ray showed decompression and fixation performed to relieve symptoms from the metastatic spinal lesion. **(B)**, Histopathology and immunophenotype of the surgical specimen showed an undifferentiated tumour on haematoxylin and eosin staining, loss of BRG1 expression, and synaptophysin positivity. Scale bars represent 20 μm. **(C)**, Serial computed tomography images showed the primary lung lesion, mediastinal lymph nodes, hilar lymph nodes, cervical lymph node, and osseous metastasis at baseline, after two cycles of treatment, and at 1-year follow-up. **(D)**, The clinical course and treatment timeline showed symptom onset, admission, surgery for symptomatic spinal metastasis, pathological diagnosis, radiotherapy plus denosumab, chemotherapy, chemo-immunotherapy, response assessment, and sintilimab maintenance. Progression-free survival exceeded 12 months at the time of manuscript submission.

To relieve neural compression, the patient underwent local decompression and spinal fixation. Subsequently, five fractions of palliative radiotherapy with a total dose of 20 Gy were administered to the metastatic lesion in the lumbar spine. After supportive treatment, his symptoms improved substantially. ECOG PS was documented as 1. Prior to the commencement of systemic anticancer therapy, the patient exhibited recurrent fever and received combination therapy with albumin-bound paclitaxel (400 mg on Day 1) and carboplatin (600 mg on Day 1) during the initial cycle. After the first cycle, the fever resolved. Thereafter, commencing with the second cycle, the patient received a combination of sintilimab (200 mg on Day 1), albumin-bound paclitaxel and carboplatin. Subsequent imaging following the second and fourth cycles indicated a partial response (PR). After the completion of four cycles of systemic therapy, the patient transitioned to maintenance therapy with sintilimab monotherapy. Denosumab was administered over an extended period throughout treatment in order to reduce the risk of skeletal-related events. No grade 3 or higher adverse events related to immunotherapy or chemotherapy were observed during the treatment period. At the time of manuscript submission, the patient’s progression-free survival (PFS) exceeded 12 months **Figure 1**.

## Discussion

This case report describes a patient with a highly aggressive SMARCA4-deficient undifferentiated tumour (SMARCA4-UT) who achieved a partial remission lasting more than 12 months following treatment with a platinum-based double-agent regimen combined with an immune checkpoint inhibitor. In addition to the favourable treatment response, this case exhibits an intriguing phenotypic feature at the pathological level: against a background of complete SMARCA4/BRG1 protein deficiency in tumour cells, isolated Syn-positive expression was observed, while other neuroendocrine markers such as INSM1 and CD56 were negative, and the histological morphology did not support a diagnosis of a classic neuroendocrine tumour. In clinical practice, such neuroendocrine-related phenotypes lacking supporting evidence are often disregarded as nonspecific staining. From a diagnostic perspective, synaptophysin positivity in an undifferentiated thoracic tumour should not be interpreted in isolation. Although it may raise the possibility of a neuroendocrine carcinoma, especially large-cell neuroendocrine carcinoma or small-cell lung carcinoma, the final diagnosis should be based on the integration of morphology, a broad immunohistochemical panel, SMARCA4/BRG1 status, and clinical-radiological findings. In addition, SMARCA4-UT may overlap morphologically with several other poorly differentiated or undifferentiated thoracic malignancies. The main differential diagnoses are summarised in **Table 1**. In the present case, the combination of complete SMARCA4/BRG1 loss, undifferentiated morphology, isolated synaptophysin positivity, absence of INSM1/chromogranin A/CD56 expression, and a dominant thoracic lesion supported the diagnosis of SMARCA4-UT rather than a conventional neuroendocrine carcinoma or another undifferentiated thoracic malignancy. However, taking into account the treatment response in this case as well as the extant literature, we hypothesize that isolated Syn positivity in SMARCA4-UT is not merely a coincidental phenomenon (3, 6, 7). Therefore, the greater significance of this case lies in proposing a hypothesis worthy of further investigation: whether Syn positivity indicates a specific differentiation tendency or treatment-related significance.

**Table 1:** Key differential diagnoses of synaptophysin-positive SMARCA4-deficient undifferentiated thoracic tumour.

Tumour type	Differential diagnosis from SMARCA4-UT
Large-cell neuroendocrine carcinoma (LCNEC)	LCNEC is defined by large-cell cytology, organoid/trabecular or rosette-like neuroendocrine architecture, high mitotic activity, necrosis, and expression of neuroendocrine markers such as synaptophysin, chromogranin A, or CD56. In contrast, SMARCA4-UT may show synaptophysin positivity but usually lacks well-developed neuroendocrine architecture; complete BRG1/SMARCA4 loss and absence of a broader neuroendocrine marker profile favour SMARCA4-UT (4, 8, 9).
Small-cell lung carcinoma (SCLC)	SCLC is characterised by classic small-cell cytology, nuclear moulding, scant cytoplasm, diffuse neuroendocrine marker expression, frequent TTF-1 positivity, and a very high Ki-67 index. The present tumour lacked classic small-cell morphology and showed complete BRG1/SMARCA4 loss with isolated synaptophysin positivity, arguing against SCLC (8, 10, 11).
NUT carcinoma	NUT carcinoma is an aggressive poorly differentiated carcinoma, often arising in midline thoracic sites, and may show sheets or nests of undifferentiated cells with abrupt squamous differentiation. Diffuse nuclear NUT immunoreactivity or molecular detection of NUTM1 rearrangement confirms the diagnosis; these features were not identified in the present case (12–14).
Metastatic melanoma	Metastatic melanoma may show dedifferentiated, undifferentiated, or rhabdoid morphology, thereby mimicking SMARCA4-UT. Expression of melanocytic markers such as SOX10, S100, HMB45, Melan-A, MITF, or PRAME supports melanoma; the absence of melanocytic differentiation and the presence of complete BRG1/SMARCA4 loss favoured SMARCA4-UT in this case (15–18).
Malignant mesothelioma	Malignant mesothelioma is suggested by pleural-based growth and epithelioid, sarcomatoid, or biphasic morphology, supported by mesothelial marker expression and/or BAP1 or MTAP loss. The present case showed a dominant pulmonary lesion with undifferentiated morphology and complete BRG1/SMARCA4 loss rather than a pleural-based mesothelial tumour (19–21).
Primary or metastatic thoracic sarcoma	Thoracic sarcomas are heterogeneous mesenchymal tumours with spindle-cell, epithelioid, pleomorphic, or rhabdoid morphology and lineage-dependent immunophenotypes. Although SMARCA4-UT may appear sarcoma-like, lack of specific mesenchymal differentiation, smoking-associated thoracic presentation, and complete BRG1/SMARCA4 loss support SMARCA4-UT rather than a true sarcoma (22–24).
Metastatic SMARCA4-deficient tumour from an extrathoracic site	Extrathoracic SMARCA4-deficient undifferentiated tumours may arise in the ovary, uterus, gastrointestinal tract, sinonasal tract, kidney, or other sites and can share rhabdoid or undifferentiated morphology with BRG1/SMARCA4 loss. In the present case, clinical history, imaging distribution, and the dominant thoracic lesion favoured primary thoracic SMARCA4-UT over metastasis from an extrathoracic SMARCA4-deficient tumour (25–30).

Although Syn positivity is one of the markers associated with neuroendocrine tumours, it does not support the presence of neuroendocrine differentiation in Syn-positive SMARCA4-UT. Moreover, given the high degree of context-dependence of epigenetic regulation, the neuroendocrine maintenance mechanisms identified in small cell lung cancer cannot be directly extrapolated to SMARCA4-UT, which often originates from non-neuroendocrine epithelium (31). Furthermore, as the core ATPase of the chromatin remodelling complex, SMARCA4/BRG1 plays a critical role in maintaining transcriptional stability and enhancer barrier function in the non-neuroendocrine lineage; its loss can lead to a weakening of the original lineage-specific constraints (32). In this context, “positive” SYN expression is more likely to result from the abnormal retention or passive de-repression of certain neuroendocrine-related genes rather than the reconstruction of a complete neuroendocrine transcriptional program.

While the underlying mechanism can be theoretically attributed to lineage disinhibition, the isolated expression of Syn should not be regarded as clinically insignificant nonspecific background staining. Conversely, this phenomenon may signify intratumoural biological heterogeneity, which could be associated with variations in treatment response among patients with SMARCA4-UT. Studies by Xin et al. and Junmin et al. have suggested that some Syn-positive patients with SMARCA4-UT may derive meaningful benefit from platinum-based chemoimmunotherapy (3, 6). This finding is consistent with the clinical course and prognostic characteristics observed in the present case.

Several limitations should be acknowledged. As a single-patient case report, this case cannot establish a causal association between isolated Syn positivity and benefit from chemoimmunotherapy. The proposed link between SMARCA4 loss and Syn expression remains speculative, as mechanistic experiments and genomic or epigenomic validation were not performed. Given the rarity and heterogeneity of SMARCA4-UT, these diagnostic and therapeutic implications should be interpreted cautiously and may require larger-scale studies for further validation.

## Conclusion

We report a case of Syn-positive SMARCA4-UT that achieved a durable partial response lasting more than 12 months following treatment with a platinum-based doublet regimen combined with an immune checkpoint inhibitor. In this context, isolated Syn positivity is more likely to reflect limited lineage de-repression in the setting of SMARCA4 loss rather than true neuroendocrine differentiation.

## Data Availability

The datasets presented in this article are not readily available because the original contributions of this study are presented in this article. Further inquiries can be directed to the corresponding author. Requests to access the datasets should be directed to Wen-Jun Tang, twjlucky@163.com.
